# Supplementary material for: International fitness scale (IFIS): association with motor performance in children with obesity
Source: PeerJ. 2023 Jul 31;11:e15765. doi: 10.7717/peerj.15765 (PMC10399561; doi:10.7717/peerj.15765)
Supplement: Supplemental Information 1 — Data are reported as mean ± SD. [file peerj-11-15765-s001.docx]

**Supplementary Material**

Anthropometric measurements pre and post training in OB children.

| Table 2. Anthropometrics characteristics (n=28). | Pre-training | Post-training | *p*-value |
| --- | --- | --- | --- |
| Height (m) | 1.50 + 0.10 | 1.52 + 0.11 | 0.001* |
| Weight (kg) | 65.69 + 17.55 | 66.95 + 16.80 | 0.02* |
| BMI (kg/m^2^) | 28.86 + 4.31 | 28.60 + 3.93 | 0.35 |
| BMIz-score | 2.21 + 0.28 | 2.16 + 0.32 | 0.01* |
| BMI Percentile | 98.36 + 0.73 | 98.07 + 0.98 | 0.01* |

Data are reported as mean ± SD.
