# Supplementary material for: International fitness scale (IFIS): association with motor performance in children with obesity
Source: PeerJ. 2023 Jul 31;11:e15765. doi: 10.7717/peerj.15765 (PMC10399561; doi:10.7717/peerj.15765)
Supplement: Supplemental Information 3 [file peerj-11-15765-s003.docx]

**Codebook of IFIS outcomes (columns AB to AM)**

The International Fitness Scale (IFIS) is a questionnaire composed of five questions that summarize children/adolescents' perceived physical fitness. Each of these five questions has 5 possible answers (from the top to the bottom: Very good; Good; Average; Poor; Very poor) and each of these answers is paired with a numerical score (Very good = 5; Good = 4; Average = 3; Poor = 2; Very poor = 1). In our dataset, we reported only the numerical score for each of the questions and, since we administered the IFIS questionnaire two times (before and after the training protocol), we reported in adjacent columns before- and after-training answers to each question. The total score of the IFIS (columns AL and AM) was calculated by summing the answer to each question.

**Codebook of IFIS outcomes in the dataset**

Column AB = Your general physical fitness is: (before the training program)

Column AC = Your general physical fitness is: (after the training program)

Column AD = Your cardiorespiratory fitness (capacity to do exercise, for instance running, for a long time) is: (before the training program)

Column AE = Your cardiorespiratory fitness (capacity to do exercise, for instance running, for a long time) is: (after the training program)

Column AF = Your muscular strength is: (before the training program)

Column AG = Your muscular strength is: (after the training program)

Column AH = Your speed / agility is: (before the training program)

Column AI = Your speed / agility is: (after the training program)

Column AJ = Your flexibility is: (before the training program)

Column AJ = Your flexibility is: (after the training program)
